# Synchronously diagnosed eosinophilic granuloma and Hodgkin's disease in a 12-year-old boy: a case report

**DOI:** 10.1186/1752-1947-3-35

**Published:** 2009-01-29

**Authors:** Soheila Sarmadi, Amir B Heidari, Amir H Sina, Mohammad A Ehsani

**Affiliations:** 1 Pathology Department, Bahrami Children's Hospital, Tehran University of Medical Sciences, Tehran, Iran; 2 Pathology Department, Medical Faculty, Tehran University of Medical Sciences, Tehran, Iran; 3 Hematology and Oncology Department, Bahrami Children's Hospital, Tehran University of Medical Sciences, Tehran, Iran

## Abstract

**Introduction:**

Synchronous composite tumors are uncommon. Simultaneous, rather than metachronous or consecutive, occurrences of eosinophilic granuloma and Hodgkin's lymphoma in children are very rare. This is the first report of this kind in the medical literature.

**Case presentation:**

We report the case of a 12-year-old Iranian boy with eosinophilic granuloma localized in his leg around the knee and Hodgkin's lymphoma in a cervical lymph node. The two tumours occurred synchronously before the patient had received any treatment.

**Conclusion:**

Several cases of an association between eosinophilic granuloma and lymphoproliferative disorder have been reported. Some of these cases involve Hodgkin's lymphoma and Langerhans cell histiocytosis occurring in the same patient. Genetic or environmental etiologies have been postulated for eosinophilic granulomas which occur following Hodgkin's lymphomas, but have as yet not been proven. To our knowledge, synchronous occurrence of these two malignant processes in a patient who has not received any prior treatment is rare in children.

## Introduction

The term Langerhans cell histiocytosis (LCH) encompasses eosinophilic granuloma and two clinical syndromes: Hand-Schüller-Christian and Letterer-Siwe disease. All these diseases seem to represent similar processes in which the proliferating cells have the structural and functional features of Langerhans cells. They differ in their proliferating properties, ranging from a solitary focus (eosinophilic granuloma) to disseminated multifocal skeletal (Hand-Schuller-Christian) and disseminated multifocal skeletal and extraskeletal disease (Letterer-Siwe disease). These three basic conditions in fact represent clinical stages of the same process. This disease primarily affects young individuals during the first three decades of life. Fifty per cent of patients are in their first decade of life. The craniofacial bones are most frequently affected and other common sites include the mandible, vertebral bodies, ribs, pelvis and femur. The lesions are lytic and have sharply demarcated punched-out intramedullary defects. They are rarely intracortical and sometimes a thin sclerotic rim can be seen. Larger lesions can erode or even completely disrupt the cortex and expand into the adjacent soft tissue. In rare instances lesions have a permeative or moth-eaten appearance.

The median age for developing eosinophilic granuloma is 13 years. Pain is the most frequent initial symptom. In 5–10% of patients, general symptoms such as fever, malaise and peripheral eosinophilia may be present. Microscopic findings of Langerhans cell histiocytosis include eosinophils and Langerhans cells but only the latter specification is pathognomonic and clonal. In addition, an admixture of other inflammatory cells may be present. The proportion of Langerhans cells and inflammatory cells, especially eosinophils, can vary among different lesions and in various areas of the same lesion. Langerhans cells in a typical case are mononuclear histiocyte-like cells with oval nuclei and clearly demarcated round or oval cytoplasm. The majority of the nuclei show a prominent nuclear groove parallel to the long axis of the nucleus. Mitotic activity is typically low and occasional multinucleated forms can be seen. The most striking and distinguishing feature of these cells is their strong positivity for S-100 protein and CD1a.

## Case presentation

A 12-year-old Iranian Caucasian boy presented with a limp and bone pain in the knee region of about 2 months duration. He was otherwise relatively well and asymptomatic. There was no significant past medical history. The patient had no history of receiving chemotherapy or radiotherapy. Physical examination performed at the time of presentation revealed limitation of left knee flexion but no other abnormality on general physical examination. No lymphadenopathy or organomegaly was detected. Hematologic investigations showed hemoglobin of 11.6 g/dl with MCV = 86, platelet count of 275000/microliter and WBC of10000/microliter. ESR was 35 and biochemical test results of blood were unremarkable. Radiographic assessment of the knee revealed a lytic-sclerotic lesion on the superior part of the tibia and a bone scan showed just one bony lesion in the same area. An open biopsy was performed and the lesion was diagnosed as eosinophilic granuloma. During the 4-month follow up period, without initiation of any treatment, the patient developed constitutional symptoms of weight loss, persistent limping and bone pain in the knee region and generalized lymphadenopathy with splenomegaly noted on physical examination.

A thoracoabdominal spiral CT scan was performed and showed mediastinal, cervical, right axillary and retroperitoneal lymphadenopathies, pulmonary nodules and hepatic and splenic involvement.

The differential diagnosis was disseminated lymphomatous involvement or disseminated Langerhans cell histiocytosis. Bone marrow and cervical lymph node biopsy were performed and the initial histopathologic diagnosis of eosinophilic granuloma for bony lesion was reviewed and immunohistochemistry (IHC) staining was performed.

**Figure 1:**
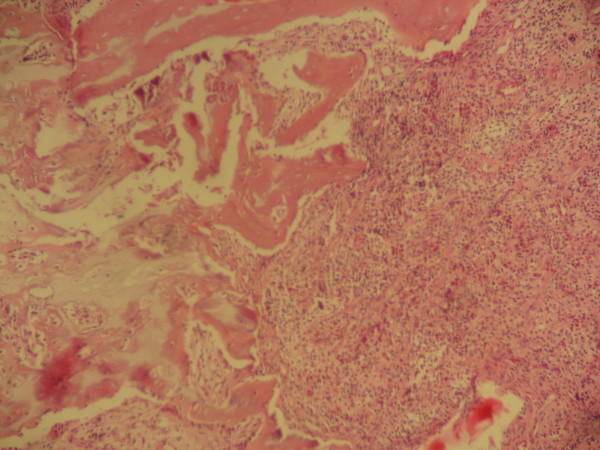
**(H&E)**. Langerhans cell histiocytosis of the bone. Collections of eosinophils and mononuclear Langerhans cells. ×10.

**Figure 2:**
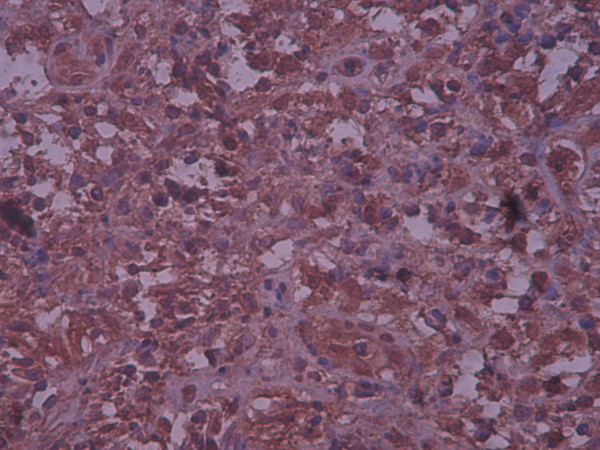
**Langerhans cell histiocytosis of the bone**. Immunohistochemical positive reaction with S100 in bone lesion ×10.

A biopsy of the lytic bone lesion revealed an admixture of inflammatory cells including many eosinophils with Langerhans cells (Figure 1). Langerhans cells showed somewhat glassy pink cytoplasms, indistinct cell borders and longitudinal coffee-bean grooves in their nuclei with undulating or indented nuclear membranes. There was no obvious mitotic activity. As a result, a pathologic diagnosis of eosinophilic granuloma was made. Subsequent bone marrow evaluation showed no abnormality (M/E = 2). Cervical lymph node biopsy revealed a lymph node with fibrotic bands, Reed Sternberg cells and Lacunar cells in a mixed inflammatory milieu which lead to a pathologic diagnosis of Hodgkin's lymphoma of the nodular sclerosis type. At this time the initial bone biopsy was reviewed to rule out any bone lymphomatous involvement by immunohistochemical staining. The immunohistochemical staining showed positive reactivity for S100 and CD1a and negative reactivity for CD15 and CD30 in large cells with folded nuclei (Figure 2).

## Discussion

Synchronously diagnosed collision tumors are considered as rare events. Langerhans cell histiocytosis (LCH) and Hodgkin's disease (HD) has been reported previously as metachronously diagnosed tumors in the literature with LCH mostly developing as a subsequent cancer in patients with HD [1-5]. One case of developing HD after LCH has also been reported [6]. Ibarrola de Andres C. et al reported simultaneous occurrence of Hodgkin's disease, Langerhans cell histiocytosis and multiple myeloma in a 35-year-old man [7] while Karadeniz G. et al reported the simultaneous development of HD and LCH in a 6-year-old boy [8] and Kjeldsberg C. R. et al described the combination of eosinophilic granuloma and malignant lymphoma in the same lymph node [5].

It is important to realize that the constellation of neoplasms might not be totally coincidental and may reflect underlying common etiologic factors as well as altered immunity as was seen in five other studies [1-4,9] in which the development of LCH was recognized after receiving chemotherapy treatment for HD. Underlying genetic susceptibilities may also play an important, although as yet not well delineated, role.

## Conclusion

We report a composite of Langerhans cell histiocytosis and Hodgkin's disease, involving different organs simultaneously in a 12-year-old boy. His past medical history showed an absence of any chemotherapy or radiotherapy which could have contributed to the evolution of Langerhans cell histiocytosis. Synchronous occurrence of HD and LCH reported here and in the study by Karadeniz G et al [8] occurring in 12 and 6-year-old boys respectively, with no history of chemotherapy, raises the possibility of a common etiologic agent which may be genetic. More immediate questions include the impact of composite tumors on the management and prognosis of the patients. Our patient was diagnosed as HD and received alternated ABVD, COPP treatment. During the 6 month follow up period all signs and symptoms, including limping and generalized lymphadenopathy, completely resolved.

Keeping in mind the probability of simultaneous occurrences of these two pathologic processes in a patient can prevent us from erroneous misdiagnoses of organ involvement by LCH. In such cases the diagnosis of synchronous HD and LCH may have a significant impact on decision making and treatment plans as well as an impact on survival.

## Abbreviations

CD: Cluster of Differentiation; CBC: complete blood count; MCV: mean corpuscular volume; WBC: white blood cells; CT scan: computed tomography scan; IHC: immunohistochemistry; M/E: myeloid/erythroid; LCH: Langerhans cell histiocytosis; HD: Hodgkin's disease; ABVD: adriamycin, bleomycin, vinblastine, dacarbazine; COPP: cyclophosphamide, concubine, procarbazine, prednisolone; ESR: Erythrocyte Sedimentation Rate; H&E: Hematoxylin and Eosin.

## Competing interests

The authors declare that they have no competing interests.

## Authors' contributions

MAE analyzed and interpreted the patient data regarding the hematologic and oncologic disease. SS and AHS performed the histological examination of the samples and were contributors in writing the manuscript. ABH obtained written informed consent from the patient's parents, carried out the literature search and produced the draft manuscript. All authors reviewed and approved the final manuscript.

## Consent

Written informed consent was obtained from the parents of the patient for publication of this case report. A copy of the written consent is available for review by the Editor-in-Chief of this journal.
